# Genetically encoded tools for tracking metabolites in live cells

**DOI:** 10.1042/BST20250115

**Published:** 2026-07-24

**Authors:** Austin H. Ablicki, Katharine L. Diehl

**Affiliations:** Department of Medicinal Chemistry, University of Utah, Salt Lake City, Utah, U.S.A.

**Keywords:** biosensors, fluorescence, fluorescence resonance energy transfer, metabolites, protein engineering

## Abstract

Biosensors enable the *in situ* measurement of metabolites in living systems over time and space. Fully genetically encoded metabolite biosensors (fGEMBs) use fluorescent proteins (FPs) linked to ligand binding domains (LBDs) to transduce the ligand binding event to a measurable change in the fluorescence behavior of the FP. Because these sensors are genetically encoded, they can be expressed in cells using standard protein expression approaches, and the fluorescence changes are quantified using fluorimetry, fluorescence microscopy, and/or flow cytometry. While there are general sensor design principles to follow, an fGEMB must be engineered for each metabolite based on a particular LBD. This development process can be slow, but there are strategies emerging to increase testing throughput and improve structure-guided design. While genetically-encoded FPs remain popular, there are now numerous chemigenetic and nucleic acid-based metabolite sensors (cGEMBs) that incorporate small molecule fluorophores. *De novo* design of LBDs is rapidly advancing as well, and the field may soon exhibit a shift away from relying on nature’s catalog of LBDs. Despite the engineering challenges, the metabolite biosensor field has expanded significantly in recent years to meet the demand for new and better-performing sensors that visualize metabolites within their cellular environments.

## Introduction

The field of biosensing can be traced back to the early 1960s when the first ‘enzyme electrode’ biosensor for glucose was developed [1]. For biosensing inside cells and tissues, the discovery of green fluorescent protein (GFP) was critical because it enabled genetic encoding of biosensor proteins with an optical readout *in situ* [2,3]. The utility of the original GFP and other fluorescent proteins (FPs) since discovered has been massively expanded by extensive engineering of physical properties, function, and color. There are now more than 1000 FP variants [4], ranging in color from purple to near-infrared (nIR) [2,3,5]. The availability of many different FP colors enabled sensors containing a donor FP and an acceptor FP able to undergo Förster resonance energy transfer (FRET). Another important development for biosensing was the circularly permuted FP (cpFP) in which the native N- and C-termini of an FP are fused and new termini are created elsewhere, allowing the cpFP to be inserted within another protein like a ligand binding domain (LBD) [3]. This design renders cpFP fluorescence sensitive to the conformation of the fusion protein, providing an optical ‘switch’ to detect conformational changes upon ligand binding [3].

A genetically encoded metabolite biosensor (GEMB) is built from a protein (or nucleic acid) that binds to the ligand of interest. Metabolite-binding proteins (MBPs) from nature, mainly bacterial transcription factors [5–20], periplasmic binding proteins, or eukaryotic G-protein-coupled receptors, have commonly been ‘repurposed’ to make GEMBs. When fused to one or more FPs, an MBP becomes a GEMB in which a conformational change in the MBP that occurs between the ligand un/bound states is ‘reported’ by the FP/s as a change in the fluorescence signal that is proportional to the amount of metabolite present. The most famous and numerous GEMBs are the Ca^2+^-sensing GECIs/GCaMPs, which are based on calmodulin [21].

The practice of using genetic encoding to ‘tag’ proteins is ubiquitous in modern biology research. But ‘tagging’ of most endogenous metabolites with fluorescent molecules in an analogous way presents a number of technical challenges, not least of which is the issue of the fluorescent tag perturbing the metabolite’s normal interactions with biomolecules. Thus, GEMBs fill an important niche, complementing other metabolite quantitation methods such as mass spectrometry and enzyme-coupled colorimetric or fluorescent assays on cell or tissue lysates [9,22,23]. When a GEMB binds its target metabolite, it produces a measurable optical signal change that then reverts back when the metabolite unbinds, allowing for a dynamic response that can track changes in the metabolite concentration over time, even in the same living cell or cell population [6,8,9,18]. A key difference between lysate-based techniques and GEMBs is that the former typically measures total metabolite (including protein bound) due to the metabolite extraction conditions [24], while GEMBs only measure the non-protein bound (‘free’) metabolite levels in live cells. The potential for these sensors to interfere with the pathways they are supposed to report on is important to consider, and GEMBs are usually engineered to minimally perturb the metabolite’s milieu, for example, by tuning the sensor’s binding affinity and/or residence time [9]. In the present mini-review, we provide an overview of how GEMBs are engineered and of how the main sensor types work.

## Fully genetically encoded GEMBs

Fully genetically encoded metabolite biosensors (fGEMBs) are typically constructed from a known MBP and one or more FPs by a structure-guided process of trial-and-error to find an orientation of the FP/s with respect to the binding domain that leads to a metabolite-induced change in fluorescence of the FP/s. This change could be an increase upon metabolite binding (‘turn-on’ sensor; e.g., increase in fluorescence intensity or FRET efficiency) or a decrease (‘turn off’ sensor; e.g., decrease in fluorescence intensity or FRET efficiency), and either direction of change is usable. The magnitude of the change is important because it, along with the apparent affinity for the metabolite, defines the dynamic range of the fGEMB, so maximizing the magnitude of the change is desirable. There are two main ways that the magnitude of the change can be improved, and these usually entail trial-and-error testing of low- to medium-throughput libraries (up to 1000s): (1) testing of various ‘linker’ amino acids added at the junction/s between the binding domain and the FP/s, which help to ‘transduce’ the conformational change to the FP chromophore and (2) random mutagenesis (via error-prone PCR, epPCR) of the fGEMB candidate. Depending on the specific properties of the MBP, mutations can be selected in a structure-guided manner and introduced to tune binding affinity and/or metabolite specificity.

The above approaches describe the most common workflows for engineering a fGEMB. While this ‘classic’ approach has yielded many useful fGEMBs, it can test only a very small subset of possible designs for a given LBD and FP/s. Several general purpose higher-throughput methods have been reported that can generate and test larger libraries (>10,000) of candidates for a given LBD [25]. For assessing candidates, there are a few examples of using *in vivo* single cell testing of bacterial libraries by fluorescence assisted cell sorting) [26–30] or single colonies on plates [31]. Another recent approach uses microfluidics to produce semi-permeable gel shell beads (GSBs) with each GSB containing the encoding DNA and expressed protein candidate for subsequent testing and identification of ‘hits’ [32]. Increasingly, computational approaches, such as molecular dynamics simulations or machine learning/AI, are being leveraged to better describe the behavior of and aid in the design of GEMBs [33–39].

### Fluorescence intensity

#### FRET

**Figure 1:**
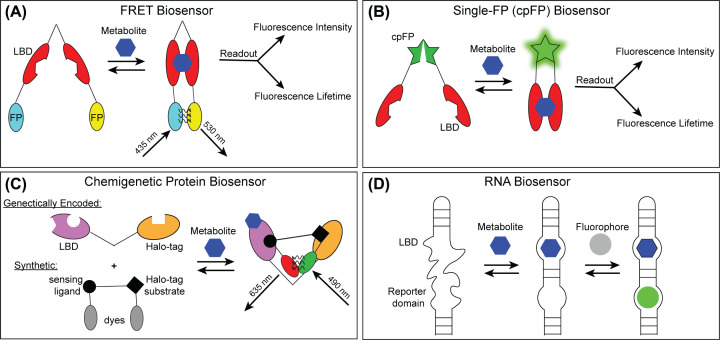
Schematics of general GEMB designs The four types of GEMBs focused on in the present review: (**A**) FRET, (**B**) single-FP (cpFP), (**C**) chemigenetic protein, (**D**) RNA. LBD = ligand binding domain, FP = fluorescent protein, FRET = Förster resonance energy transfer, cpFP = circularly permuted fluorescent protein.

FRET occurs when a donor fluorophore can transfer energy to an acceptor fluorophore. There are multiple FP FRET pairs available, although cyan and yellow FP (CFP/YFP) remain very popular [40,41]. The donor’s emission spectrum (e.g., CFP, λ_em,max_ ≈ 485 nm) must overlap with the acceptor’s excitation spectrum (e.g., YPF, λ_ex,max_ ≈ 530 nm) to enable distance-dependent energy transfer. If the FRET pair is far apart in space (>10 nm), only the donor’s emission will be detected. If the pair is close (<10 nm), FRET emission from the acceptor will be observed. The efficiency of the energy transfer increases as a function of decreasing distance between the pair, allowing for protein conformational changes to lead to a change in the FRET ratio [40–43]. Thus, FRET with FP pairs has been extensively leveraged to make fGEMBs by fusing an FP at each terminus of an LBD (Figure 1A). While FRET-based fGEMBs can be easier to initially construct, a drawback is that the FRET ratio changes are generally quite small in magnitude, limiting the dynamic range. Another disadvantage of FRET-based fGEMBs is that they are difficult to ‘multiplex’ with other FPs because of how much of the visible spectrum they require (e.g., CFP/YFP ∼400–580 nm). Even though fGEMBs that utilize an FP FRET pair have become less popular since the advent of cpFPs, new FRET-based sensors are still being reported [13,28,33,44–49].

#### Single FP (cpFP)

The GEMB field has shifted significantly toward the design of a cpFP inserted into a ligand binding protein (Figure 1B) [3,8,9,50]. Like FRET biosensors, cpFP-based biosensors rely on a conformational change in the LBD to elicit a change in fluorescence [7,9,12,19]. In this case, the conformation of the LBD perturbs the structure of the intervening cpFP and the environment of the chromophore, modulating its pK_a_, extinction coefficient, and/or quantum yield [51]. To yield a functional cpFP biosensor, substantial optimization is often needed to determine the location of the cpFP and the linkers [9]. The cpFP tends to be inserted into loops between more structured regions of the LBD, since these regions are important for ligand binding and/or conferring the conformational change [9]. The favored insertion site shows the greatest change in fluorescence intensity between -ligand and +ligand (usually reported as a fold change) [9,52]. Next, the linkers that connect the LBD and the cpFP are varied to attain fold change improvement. The linker amino acid composition and length (and insertion site) can have dramatic effects on sensor properties, including brightness, fold change, and, potentially, even specificity and affinity for the ligand [6,8–10]. Several rounds of random mutagenesis via epPCR are usually performed next to yield a biosensor with optimized properties. The cpFP-based sensors can exhibit an increase (‘turn on’) or a decrease (‘turn off’) in fluorescence intensity upon ligand binding, and the fold change magnitude ranges from about 2-fold to more than 20-fold. Interestingly, both ‘turn on’ and ‘turn off’ sensors can derive from the same LBD depending on the insertion site and/or linkers [8]. Compared to FRET sensors, the magnitude of the signal change possible with a cpFP-based fGEMB is much larger, leading to a larger dynamic range. Since the signal from the cpFP-based fGEMBs is an intensity change, this change must be normalized to account for changes in the sensor concentration or other factors that modulate fluorescence intensity but are ligand independent. This normalization is performed in two main ways: (1) by measuring the fluorescence emission at two excitation wavelengths of the cpFP, one that changes in a ligand-dependent fashion and one that is ligand independent or (2) by appending a second FP to one of the termini of the fGEMB, the fluorescence behavior of which is ligand independent. This second ligand-independent measurement enables a ratiometric output akin to how FRET is calculated. While this necessity to measure two different FPs at different wavelengths recalls a disadvantage of the FRET fGEMB format and the FPs will have different chromophore maturation rates [53], there is modularity in this format in terms of selecting a ligand-independent FP. One area of development is to employ nIR cpFPs, and there are a number of such fGEMBs just being reported [5,6,10,31]. While the nIR FPs require higher laser power due to decreased quantum yields, excitation at >600 nm offers lower phototoxicity and background (e.g., from autofluorescence) for tissue and live animal imaging [5,6,10,31].

The field of cpFP biosensors has expanded greatly in the past few years. There has been an explosion of new fGEMBs reported for metabolites such as pyruvate [52], acetyl-CoA [9], D2HG [7,12], NAD^+^ [19], NADP+/NADPH [54], malonyl-CoA [9], arginine [55], methionine [56], glutamate [57], and glutamine [17,58].

### Fluorescence lifetime imaging microscopy

In fluorescence intensity imaging measurements, the image is composed of pixels, each representing the magnitude of the light emitted by the fluorophore upon excitation [59]. With fluorescence lifetime imaging microscopy (FLIM), the pixels in the image represent time, i.e., the fluorescence lifetime for the fluorophore, not intensity [13,59–61]. An advantage of FLIM for metabolite quantitation by GEMBs is that the signal is theoretically independent of the fluorophore/FP concentration. FLIM does not require ratiometric measurement, but it does require calibration steps [59,62]. Microscopes that can measure fluorescence lifetime are not as widely available or easy to use as standard fluorescence microscopes, and FLIM data analysis is complex [59]. For fGEMBs, specific FPs that have been engineered to have a long ‘baseline’ fluorescence lifetime tend to work best for FLIM. FLIM is typically slower than fluorescence imaging because sufficient photons must be collected for accurate fitting to determine the fluorescence lifetime parameter for each pixel. FLIM of fGEMBs, like intensity-based measurement, is influenced by pH, autofluorescence, and other factors posed by the specific cellular microenvironment of the sensor. FLIM-based fGEMBs are a growing area that will continue to benefit from advances in FPs and in FLIM methodology.

#### FRET-FLIM

In FRET, the donor fluorophore undergoes fluorescence decay after excitation, and the time it takes to decay back to the ground state is altered by the presence of the acceptor fluorophore [40,59]. When the FRET acceptor is close enough to the donor in space (<10 nm), the fluorescence lifetime of the donor decreases [40,59]. Thus, the change in the fluorescence lifetime of the donor of a FRET-based fGEMB can be measured by FLIM to quantify the sensor’s target metabolite [59]. Even though the acceptor FP is not directly measured in FLIM, the experiment still requires both FPs, which can pose challenges for multiplexing [59]. Differences in maturation rate between the donor and acceptor FP can also still be confounding with FRET-FLIM. One recent example of a new FRET-FLIM fGEMB is for NAD^+^ [63].

#### Single FP-FLIM (cpFP-FLIM)

Single FP FLIM biosensors have the same design as the fluorescence intensity version but are usually constructed from specific cpFPs that have suitable fluorescence lifetime properties, although some fGEMBs can be used with both FLIM and intensity measurements [10]. Akin to the intensiometric fGEMBs, the fluorescence lifetime of the FP could increase or decrease as a function of the metabolite [13]. As described above, cpFP-FLIM does not require ratiometric normalization, giving it high multiplexing potential, but it is important to note that cpFP-FLIM biosensor engineering is still at least as challenging as for the intensiometric cpFP fGEMBs. fGEMBs designed for cpFP-FLIM are becoming increasingly popular, and there are numerous recent examples of new such sensors: ATP [13,64], glucose [13], citrate [13], cAMP [13], lactate [10,32], Ca^2+^ [61,65,66], histidine [67], and acetylcholine [68].

## Chemigenetic GEMBs

Chemigenetic (aka, semi-synthetic) GEMBs (cGEMBs) utilize one or more synthetic dyes. This format takes advantage of the often-superior properties of small molecule fluorophores compared to FPs (e.g., pH insensitivity, brightness). It also enables the use of metabolite-binding nucleic acid aptamers to construct biosensors. The synthetic dye part of the cGEMB must be delivered inside the cell to ‘meet up’ with the genetically encoded part to assemble the complete biosensor, which poses challenges such as procedural complexity, inefficient delivery, and assembly kinetics.

### Protein based

These metabolite biosensors utilize a protein domain that binds the metabolite of interest, but they incorporate small molecule fluorophores [63,69–74]. The most popular approach for conjugating the dye/s to the LBD is to use protein labeling tags, e.g., SNAP-tag, Halo-tag [16,70,72–74]. The dyes (synthetic only or a combination of synthetic dye and FP) undergo a fluorescence change, most commonly via FRET or quenching [5,57,75], due to the change in conformation of the LBD that changes the spatial orientation of the dye/s (Figure 1C). There are now an array of cGEMB designs [76]. One example is a recently reported ‘fluorogenic, rhodamine-based, chemigenetic biosensor’ (FOCS) for NADPH [73]. The genetically encoded part is an NADPH binding domain linked to a Halo-tag. The Halo-tag tethers a set of rhodamine dyes that undergo FRET only when NADPH is bound to the sensor, giving this sensor very low background. Another design uses photoinduced electron transfer quenching instead of FRET [74,75]. An LBD-Halo-tag fusion is used to couple a rhodamine dye, and a tryptophan (Trp) within the Halo-tag is positioned close in space to the rhodamine such that the Trp quenches the dye when the ligand is absent. When the ligand binds to the sensor, the Trp moves away from the dye, eliminating the quenching.

### Nucleic acid-based

While proteins have dominated the metabolite biosensor field, RNA aptamers that bind to a metabolite of interest have also been used to construct biosensors [77,78], and these sensors are increasing in their popularity. These sensors consist of an RNA aptamer with two domains: (1) a sensing domain that binds the ligand of interest and (2) a reporter domain that binds to a fluorogenic dye. The RNA domains are oriented such that, when the ligand binds to the sensing domain, a conformational change occurs that modulates whether a fluorogenic dye can bind to the reporter domain or not (Figure 1D). This class of biosensors has taken off over the last several years, but some issues remain with the fluorogenic dye substrates, such as low photon yield and poor permeability [79]. Two recent examples are a ratiometric dual-metabolite biosensor that measures the cyclic di-GMP/cGAMP ratio [80] and a glycine biosensor [81]. With the cyclic di-GMP/cGAMP sensor, a riboswitch for each metabolite with its reporter domain is linked via a central tRNA scaffold. One sensor lights up red, and the other lights up green, yielding the ratio of the two metabolites from a single cGEMB.

## Discussion

**Figure 2:**
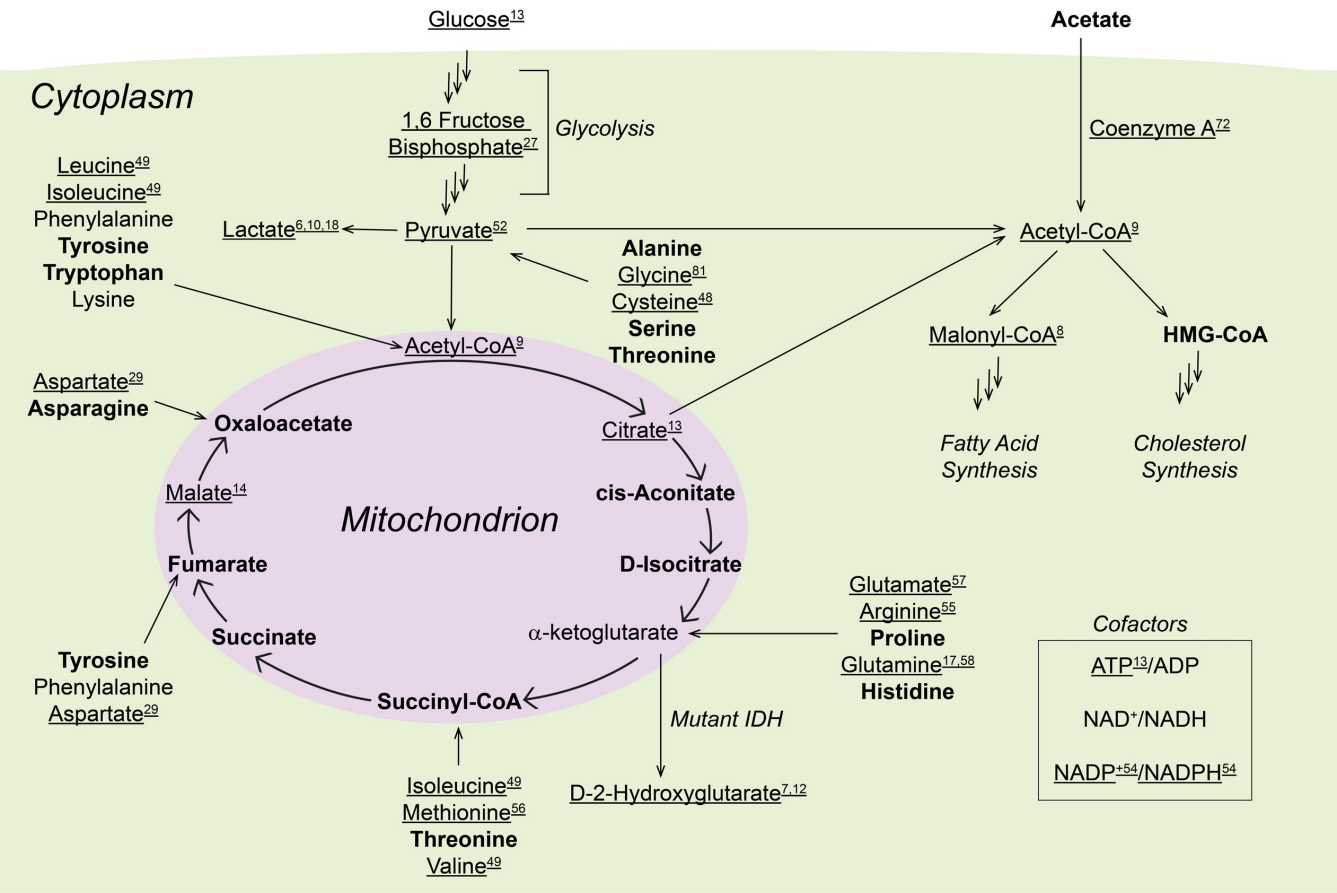
Coverage of central carbon metabolism by GEMBs A simplified overview of carbon metabolism that highlights metabolites with no biosensor yet reported in **bold font**. Metabolites for which a new biosensor has been reported within the past 5 years are underlined and with respective citations shown as superscripts. Metabolites for which the last biosensor was reported more than 5 years ago are shown with regular font and no citation.

There are estimated to be over 200,000 human metabolites [82,83]. About 20,000 of these have been directly detected in human samples, which is similar to the total number of protein-coding genes in the human genome [84]. Thus, there are biosensors available for less than 1% of the detected human metabolome, and incredible opportunities remain for making new GEMBs (Figure 2). The challenge lies in the time and effort required to engineer the first generation of a biosensor for a given metabolite, as well as subsequent versions of the same base biosensor with specific properties to make it suitable for particular applications (e.g., different ligand affinities, FPs of different colors or with different properties).

Two of the major engineering challenges for LBDs are a lack of *a priori* knowledge of protein ligand binding behavior and sufficient selectivity for structurally similar metabolites. It is estimated that nature has produced billions of unique proteins [85], but only a small fraction has been characterized [84]. GEMB developers co-opt naturally occurring LBDs, so the degree of selectivity of different GEMBs varies depending on what evolution produced. For example, there is a naturally evolved bacterial LBD that is highly selective for malonyl-CoA [8,16], but the as-yet characterized LBDs for coenzyme A (CoA) and other acyl-CoAs have only low to moderate selectivity [9,72,86,87]. While directed evolution can be used to improve LBD selectivity [88,89], the GEMB field has not yet made a concerted effort to do so, instead focusing on engineering affinity, responsivity, and different FPs/dyes. Importantly, there is no quantitative answer for what is ‘selective enough’ for a given metabolite, and our knowledge of intracellular/organellar metabolite concentrations is poor, making it difficult to pinpoint selectivity and affinity requirements. *De novo* protein design is beginning to expand LBDs beyond what nature has to offer [90–95]. There are now ‘artificial’ LBDs for farnesyl pyrophosphate [90], cortisol [94], 17-α-hydroxyprogesterone [94], and several drugs, e.g., apixaban [91,94]. Efforts to characterize the ligand binding behavior across the proteome (e.g., AlphaFill) [96] and of libraries of designed proteins [92,93] will make it easier to find and design suitable LBDs for GEMB development. Computational design of ligand-induced protein-protein interactions have even made metabolite-induced dimerization biosensors [90,94,95]. While this approach has not yet been used to make a fluorescent GEMB, these ‘artificial’ LBDs could be made into GEMBs using the same approach as for ‘natural’ LBDs. For nucleic acid-based sensors, evolution of aptamer ligand binding by SELEX yielded *de novo* RNA aptamers that bind to leucine [97], rATP [98], and several drugs [97,99] that could be made into cGEMBs. With respect to the fluorescent part, engineering of FPs with an array of valuable properties continues apace [100,101], and advances in chemigenetic sensor designs further expand the functionality by utilizing fluorescent dyes [76]. For combining these two parts to make a functional GEMB, structural approaches can guide design, but there is yet no substitute for trial-and-error testing of candidate designs, whether libraries of dozens or millions are needed to achieve the desired GEMB.

Among the modalities, intensiometric cpFP fGEMBs’ good sensitivity and user-friendliness (e.g., easy to express in cells, compatible with common microscopes, simple data analysis), make them the best general choice, in our opinion. However, we note that the existing data for head-to-head experimental comparisons of different GEMBs [61,102,103] are sparse, making it often difficult to compare sensor performance in the cell context based on literature. Moreover, the GEMB engineering process is based on many assumptions about the cell environment that may not be accurate (e.g., metabolite concentrations, sensor folding/maturation behavior, pH). The best GEMB is the one that a user can successfully deploy in their application of interest. A user should consider if the delivery modality is compatible with their system (e.g., fGEMB vs cGEMB, organelle), and if they have the necessary microscope (e.g., intensity vs FLIM, wavelengths needed). We suggest selecting the GEMB that has been demonstrated already in a similar application (e.g., cell type, organelle, timeframe, perturbation). However, given the incredible number of unknown variables that could influence GEMB performance in a particular application/context, well-designed experimental controls are critical for interpreting GEMB data appropriately. Current GEMBs are better at measuring relative metabolite levels between experimental conditions than absolute quantitation, so making the right comparison is critical for success.

Developing a GEMB is similar to designing a space probe in the sense that we have limited knowledge about the alien environment that the probe will encounter once deployed. We must make many assumptions about the inner workings of the cell that, if wrong, compromise our interpretation of the probe’s signal. Despite the challenges, the impetus for making new and improving existing GEMBs is the incredible complexity of metabolism and the extraordinary promise of GEMBs to report to us on the activity of small molecules from within living cells and tissues.

## Perspectives

- Importance: GEMBs enable real-time, compartmentalized tracking of metabolites in live cells, whereas other methods are limited to static measurements in cell lysates.
- Current Thinking: While some GEMB developers focus on expanding the biosensor toolkit for one metabolite, others work on making the first GEMB for metabolites that do not yet have one.
- Future Direction: Advancements in protein/nucleic acid design and engineering will accelerate the proliferation of GEMBs, but while many of these new GEMBs will enable previously inaccessible applications, users may find it increasingly challenging to navigate the growing array of options.

## Open Access

Open access for this article is provided via our Subscribe to Open programme, made possible through the support of our subscribing institutions. Learn more here: https://portlandpress.com/pages/open_access_options_and_prices#conditional.

## Abbreviations

CFP: cyan FP
CoA: coenzyme A
cGEMB: chemigenetic GEMB
cpFP: circularly permuted FP
fGEMB: fully genetically encoded metabolite biosensor
FLIM: fluorescence lifetime imaging microscopy
FRET: Förster resonance energy transfer
GEMB: genetically encoded metabolite biosensor
GFP: green fluorescent protein
GSB: gel shell bead
MBP: metabolite-binding protein
nIR: near-infrared
YFP: yellow FP
